# Relationship between Fibroblast Growth Factor 23 and Biochemical and Bone Histomorphometric Alterations in a Chronic Kidney Disease Rat Model Undergoing Parathyroidectomy

**DOI:** 10.1371/journal.pone.0133278

**Published:** 2015-07-17

**Authors:** Hung-Wei Liao, Peir-Haur Hung, Chih-Yen Hsiao, Hung-Hsiang Liou, Hsin-Shih Lin, Tsang-Hai Huang, I-Ming Jou, Kuen-Jer Tsai

**Affiliations:** 1 Jia-yi Clinic, Taoyuan, Taiwan; 2 Department of Internal Medicine, Ditmanson Medical Foundation Chia-yi Christian Hospital, Chiayi City, Taiwan; 3 Department of Applied Life Science and Health, Chia-Nan University of Pharmacy and Science, Tainan, Taiwan; 4 Division of Nephrology, Department of Medicine, Hsin-Jen Hospital, New Taipei City, Taiwan; 5 Institute of Physical Education, Health and Leisure Studies, National Cheng Kung University, Tainan, Taiwan; 6 Department of Orthopedics, College of Medicine, National Cheng Kung University, Tainan, Taiwan; 7 Institute of Clinical Medicine, College of Medicine, National Cheng Kung University, Tainan, Taiwan; 8 Center of Clinical Medicine, National Cheng Kung University Hospital, College of Medicine, National Cheng Kung University, Tainan, Taiwan; National Yang-Ming University, TAIWAN

## Abstract

**Background:**

Phosphate burden in chronic kidney disease (CKD) leads to elevated serum fibroblast factor-23 (FGF-23) levels, secondary hyperparathyroidism and chronic kidney disease-mineral bone disorder (CKD-MBD). However dissociated hyperphosphatemia and low serum FGF-23 concentrations have been observed in experimentally parathyoridectomized rats. The relationships between serum mineral, hormone, and bone metabolism may be altered in the presence of CKD. The aim of our study was to investigate whether a consistent relationship existed between serum FGF-23 levels, specific serum biochemical markers, and histomorphometric parameters of bone metabolism in a parathyroidectomized CKD animal model.

**Results:**

Sprague Dawley rats were divided into 3 groups: parathyroidectomy (PTX) and CKD (PTX+CKD, 9 rats), CKD without PTX (CKD, 9 rats), and neither PTX nor CKD (sham-operated control, 8 rats); CKD was induced by partial nephrectomy. At 8 weeks after partial nephrectomy, serum biomarkers were measured. Bone histomorphometries of the distal femoral metaphyseal bone were analyzed. The mean serum FGF-23 levels and mean bone formation rate were the highest in the CKD group and the lowest in the PTX+CKD group. Bone volume parameters increased significantly in the PTX+CKD group. Pearson’s correlation revealed that serum FGF-23 levels associated with those of intact parathyroid hormone, phosphate, collagen type I C-telopeptide, and calcium. Univariate linear regression showed that serum FGF-23 values correlated with bone formation rate, bone volume, and osteoid parameters. Stepwise multivariate regression analysis revealed that circulating FGF-23 values were independently associated with bone volume and thickness (β = -0.737; p < 0.001 and β = -0.526; p = 0.006, respectively). Serum parathyroid hormone levels independently correlated with bone formation rate (β = 0.714; p < 0.001) while collagen type I C-telopeptide levels correlated with osteoid parameter.

**Conclusion:**

Serum FGF-23 levels independently correlated with bone volume parameters in rats with experimentally induced CKD.

## Introduction

In chronic kidney disease, CKD-mineral and bone disorder (CKD-MBD) is a disturbance in mineral metabolism and bone remodeling. Subsequently, vascular calcification may develop. CKD-MBD affects many patients who have CKD and increases their morbidity and mortality [1–4]. The onset of CKD-MBD is considered to be caused by an abnormality in mineral metabolism when renal function declines. Subsequently, hormone dysregulation, osteodystrophy, and cardiovascular complication appear.

Fibroblast growth factor23 (FGF-23) is a regulator of phosphate metabolism and is elevated in patients with CKD [5]. The hormone FGF-23 is derived mainly from osteocytes in bone [6,7] and acts on proximal renal tubules to maintain serum phosphate homeostasis [8,9] by excreting excess phosphate through the kidney [10,11]. In CKD, phosphate retention occurs as functional renal mass diminishes; this stimulates FGF-23 synthesis to increase renal excretion of excess phosphate [12]. Although disrupted phosphate homeostasis in patients with CKD also induces secondary hyperparathyroidism, the elevation of serum FGF-23 occurs earlier than that of serum PTH levels [13,14].

Since FGF-23 regulates serum mineral homeostasis and derives from bone, FGF-23 has effect on bone metabolism. It has been suggested that FGF-23 is directly associated with bone metabolism. Treatment with resorption inhibitors or anabolic agents could modulate bone formation rate and simultaneously influence circulating FGF-23 concentrations [15]. In patients with CKD, hyperphosphatemia and secondary hyperparathyroidism lead to a high turnover of bone disease [16,17]. Since serum FGF-23 concentrations concurrently rise in these patients, a high turnover bone disease may accompany high levels of FGF-23 [18]. Furthermore, an independent negative association between FGF-23 and bone mineral density at the total hip and femoral neck has also been observed in CKD stage 4 patients [19].

Nevertheless, changes in serum FGF-23 levels are not always associated with changes in serum phosphate concentration, according to results from animal studies [20]. In rodents subjected to parathyroidectomy (PTX), a dissociated change in serum phosphate and FGF-23 levels was observed. This dissociation makes the principle that chronic phosphate burden in CKD resulting in increased serum PTH and FGF-23 levels and leading to renal osteodystrophy may not be consistent when present in PTX animals accompanied by renal failure. As a result, the relationship between hormone levels and bone metabolism may be altered. We questioned whether serum FGF-23 levels correlated with changes in parameters of bone metabolism when a model included underwent PTX and renal failure animals.

Therefore, we designed an experimental CKD model combined with PTX in which serum phosphate and FGF-23 levels were dissociated. We investigated the relationship between FGF-23, biochemical markers, and bone histomorphometric parameters in this model with the aim of providing more information concerning hormone and bone metabolism in CKD.

## Materials and Methods

### Animals

Six-week-old male Sprague Dawley rats were housed under controlled conditions (room temperature, 22°C ± 1°C; alternating 12-h light and dark periods). All animals were given rat chow (Purina Rodent Chow 5001, Labdiet, Richmond, IN) containing 0.95% calcium and 1.07% phosphate (weight/weight dry food) and tap water *ad libitum* throughout the study. The National Cheng Kung University Animal Ethics Committee approved all of the experimental procedures (Permit Number: 100269). The care and handling of the animals were in accordance with the National Institute of Health guidelines for ethical treatment of animals. All surgery was performed under anesthesia, and all efforts were made to minimize suffering.

### Experimental Procedure

After the rats were acclimatized to the laboratory environment for 1 week, they were randomly divided into 3 groups: PTX and CKD (PTX+CKD, 9 rats), CKD without PTX (CKD, 9 rats), and neither PTX nor CKD (sham-operated control, 8 rats). The PTX+CKD group underwent PTX caused by electrocautery under a dissecting microscope [21,22] after anesthesia. Anesthesia was induced with 5% isoflurane mixed with 70% N_2_O and 30% O_2_ in an induction chamber and regulated as necessary. Surgical-depth anesthesia was maintained with 1.5% isoflurane. The parathyroid glands of the other rats (CKD and sham-operated control) were exposed, but electrocautery was not performed. In all 9 PTX+CKD rats, blood samples withdrawn 3 days after PTX showed serum calcium levels < 6.0 mg/dL that were confirmed successful PTX [23].

For the PTX+CKD rats (1 week after PTX) and CKD rat models, CKD was induced by a partial nephrectomy performed as a 2-step procedure with general anesthesia as described previously [21–23]; in sham-operated control rats, the kidneys were exposed but no nephrectomy was performed. At 8 weeks after induction of CKD, blood samples were obtained by intracardiac puncture (all 26 rats) after general anesthesia, spot urine samples were collected (feasible only in 19 rats), and the rats were euthanized. Serum was separated immediately by centrifugation (1500×*g* for 15 min) and stored at −75°C for future assays.

### Blood and Urine Assays

Serum was analyzed for blood urea nitrogen (BUN), creatinine (Cr), phosphate, calcium, and alkaline phosphatase (ALP) levels with an automatic chemistry analyzer. Enzyme-linked immunosorbent assay kits were used to measure levels of serum intact parathyroid hormone (PTH,ALPCO, Salem, NH), FGF-23 (Kainos Laboratories, Tokyo, Japan), osteocalcin (Biomedical Technologies, Inc., Stoughton, MA), 1,25-dihydroxyvitamin D_3_ (1,25-(OH)_2_D_3_) (MyBioSource, Inc., San Diego, USA) and collagen type I C-telopeptide (CTX) (MyBioSource, Inc., San Diego, USA) levels. Urine chemistry values were measured with an automatic chemistry analyzer. Urinary fractional excretion of phosphate was calculated as (urine phosphate × serum creatinine) × [100/(serum phosphate × urine creatinine)]. The same equation was applied to calculate urinary fractional excretion of calcium.

### Bone Histomorphometry

The rats were injected intraperitoneally with calcein (20 mg/kg) (Sigma-Aldrich, St Louis, MO) 10 days and 3 days before being euthanized. After death, the distal femurs were excised, placed in 70% ethanol, and dehydrated. Specimens were embedded in methyl methacrylate according to the manufacturer (Fluka, 64200, Sigma-Aldrich, St Louis, MO) and not decalcified. Longitudinal sections (thickness, 5μm) were made in the sagittal plane with a motorized microtome (Microm HM 355S, Microm International GmbH, Walldorf, Germany) and stained with Masson trichrome.

Quantitative study of the distal femoral metaphysis was performed according to histomorphometric procedures as described previously [24–26] and evaluated using image analysis software (Image Pro Plus 6.1 for Windows; Media Cybernetics, Silver Spring, MD). The bone turnover, mineralization, and volume (TMV) classification, suggested by the Kidney Disease Improving Global Outcomes (KDIGO) guidelines, was used to assess renal osteodystrophy [27]. Volume histomorphometric parameters were measured including bone volume ratio (BV/TV) (%), trabecular thickness (μm), trabecular separation (space between trabeculae, μm), and trabecular number (1/mm). The femoral sections were photographed under a fluorescent light microscope (×200) using a digital camera (COOLPIX 4500; Nikon, Tokyo, Japan). Osteoid parameters were measured as osteoid volume ratio (1) (OV/BV) = osteoid volume/bone volume; (2) osteoid surface ratio (OS/BS) = osteoid surface/ bone surface; and (3) osteoid thickness (O.Th) = (osteoid area/osteoid perimeter) x 2/1.2 [25]. Dynamic histomorphometric parameters were determined from the metaphysis, including (1) percent mineralized bone surface (MS/BS) (%) = ([perimeter of a single labeled surface × 0.5] + [perimeter of a double labeled surface])/(perimeter of the total bone surface); and (2) mineral apposition rate (MAR) (μm/d) = (distance between the 2 fluorescent labels)/(time between the 2 injections of the fluorescent label) x 1.2 [25]. Bone turnover was determined using bone formation rate (BFR) (μm/d) = percent mineralized bone surface × mineral apposition rate. Assessment of mineralization was evaluated by osteoid maturation time (OMT) = O.Th/MAR.

### Statistical Analysis

Data analysis was performed with statistical software (SPSS, version 17.0, IBM Corporation, Armonk, NY). Data on figure were presented with mean ± SEM. The others were reported as mean ± SD for continuous variables. Variables those without normal distribution were analyzed after logarithmic transformation for normal distribution. ANOVA and post hoc Games-Howell test were used to compare difference between groups. The relations between serum biomarkers were evaluated using Pearson’s correlation. Univariate regression analysis was performed to assess linear relationships between serum biomarkers and bone parameters. Stepwise multivariate regression analysis was performed to determine the variables had independent association with bone histomorphometric parameters. The probability of entry was 0.05 and removal was 0.1 in stepwise analysis. Two sided p value < 0.05 was considered statistically significant.

## Results

### Assessment of serum biochemical parameters

**Fig 1:**
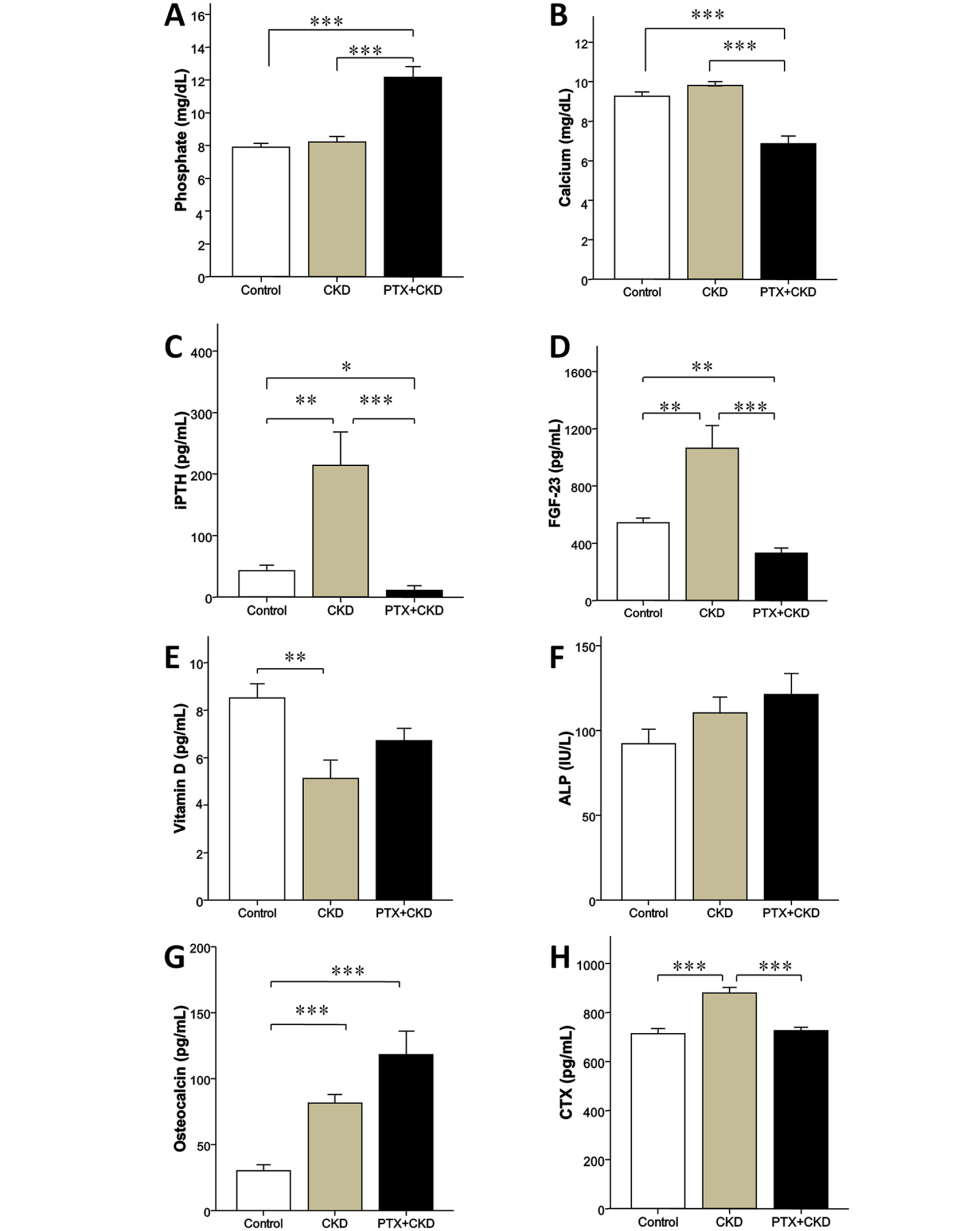
Comparison of serum biochemical parameters between control CKD and PTX +CKD group. (A) Serum phosphate levels. (B) Serum calcium levels. (C) Serum intact PTH levels. (D) Serum FGF-23 levels. (E) Serum Vitamin D (1,25-(OH)_2_D_3_) levels. (F) Serum ALP levels. (G) Serum osteocalcin levels. (H) Serum CTX levels. n = 8 control, n = 9 CKD, n = 9 PTX +CKD. Results were presented as mean ± SEM. * p < 0.05, ** p < 0.01, *** p < 0.001.

The severity of renal failure was similar between the PTX+CKD and the CKD groups (BUN: 61 ± 26 and 68 ± 26 mg/dL, respectively; Cr: 1.2 ± 0.4 and 1.2 ± 0.4 mg/dL, respectively). The mean BUN and Cr levels of the sham-operated control group (BUN: 18 ± 3 mg/dL; Cr: 0.5 ± 0.1 mg/dL) were significant difference from PTX+CKD and CKD groups. (all p **<** 0.001). The mean serum phosphate levels were significantly higher, and calcium levels were significantly lower in the PTX+CKD animals compared to either the CKD or control groups (Fig 1). The mean serum phosphate and calcium levels were similar between the CKD and sham-operated groups (Fig 1).

### Fractional Excretion of Electrolytes

The mean fractional excretion of phosphate was significantly greater in the CKD group than in controls (20 ± 10% and 6 ± 3%, respectively; p = 0.04); there was no significant difference in fractional excretion of phosphate between control and PTX+CKD groups (7 ± 6%) or between the CKD and PTX+CKD groups. Referred to mean fractional excretion of calcium, there was significantly greater (p = 0.01) in the PTX+CKD group (4 ± 3%) than in the control group (0.2 ± 0.1%). But the fraction excretion of calcium in the CKD group (4 ± 4%) was not different to the other two groups.

### Circulating Intact PTH Levels

Mean serum intact PTH level was significantly greater in the CKD than in the control group and significantly less in the PTX+CKD compared to the control or CKD group (Fig 1). These PTH levels confirmed the presence of PTX in the PTX+CKD group (lowest PTH levels) and secondary hyperparathyroidism in the CKD group (highest PTH levels).

### Assessment of Serum Biomarkers of Bone

Evaluation of bone biomarkers showed that mean ALP was similar for the control, CKD, and PTX+CKD groups (Fig 1). The mean serum osteocalcin level was greater in the CKD and PTX+CKD groups than in the control group. The mean serum FGF-23 levels were significantly different between the 3 groups, being greatest in the CKD group and lowest in the PTX+CKD group. The mean 1,25-(OH)_2_D_3_ levels in the CKD group was significantly lower than in the control group, while mean serum levels of CTX, a bone resorption marker [28], were significantly higher in the CKD group than in the control and PTX+CKD groups. The bone resorption activity was highest in CKD group according to the serum CTX data.

### Histomorphometric Bone Parameters

Evaluation of static histomorphometric parameters showed that the mean bone volume ratio and mean trabecular number were significantly greater in the PTX+CKD group than in the control or CKD groups. Mean trabecular thickness was significantly greater, and mean trabecular separation was significantly lower in the PTX+CKD compared to the CKD group (Table 1). Osteoid thickness in the CKD group was significantly greater than in the PTX+CKD group (Table 1), but no other osteoid parameter was significantly different between the groups.

**Table 1:** Structural and Dynamic Histomorphometric Parameters of Experimental Chronic Kidney Disease Rats that did or did not Undergo Parathyroidectomy.*

Parameter	Sham-operated Control	Chronic Kidney Disease (CKD)	Parathyroidectomy and Chronic Kidney Disease (PTX+CKD)
No. rats	8	9	9
**Structure parameters**			
Bone volume ratio	19 ± 5	15 ± 4	31 ± 8^a^ ^,^ ^b^
Trabecular thickness (μm)	88 ± 27	60 ± 13	91 ± 21^b^
Trabecular separation (μm)	624 ± 258	777 ± 231	395 ± 200^b^
Trabecular number (1/mm)	1.5 ± 0.8	1.3 ± 0.6	2.4 ± 0.8^a^ ^,^ ^b^
**Dynamic parameters**			
MS/BS (%)	26 ± 5	35 ± 10	21 ± 7^b^
MAR (μm/d)	1.8 ± 0.38	2.5 ± 0.49^c^	1.3 ± 0.43^b^ ^,^ ^c^
BFR (μm/d)	0.5 ± 0.1	0.9 ± 0.2^a^	0.3 ± 0.2^b^ ^,^ ^c^
**Mineralization**			
OV/BV	6.7 ± 6.7	15.0 ± 15.0	4.0 ± 2.7
OS/BS	19.3 ± 16.3	28.9 ± 25.5	9.9 ± 5
O.Th	24.8±6.2	38.9 ± 14.7	19.7 ± 6.4^d^
OMT	14.4± 5.8	15.4 ± 3.3	16.9 ± 8.2

*N = 26 rats. Data reported as mean ± SD. Abbreviations: CKD, chronic kidney disease; PTX+CKD, parathyroidectomy and chronic kidney disease; MS/BS, percent mineralized bone surface; MAR, mineral apposition rate; BFR, bone formation rate; OV/BV, osteoid volume ratio; OS/BS, osteoid surface ratio; O.Th, osteoid thickness; OMT, osteoid maturation time

^a^p < 0.01, compared with sham-operated control group.

^b^p < 0.01, compared with CKD group.

^c^p < 0.05, compared with sham-operated control group.

^d^p < 0.05, compared with CKD group. NS, no significant (p > 0.05)

**Fig 2:**
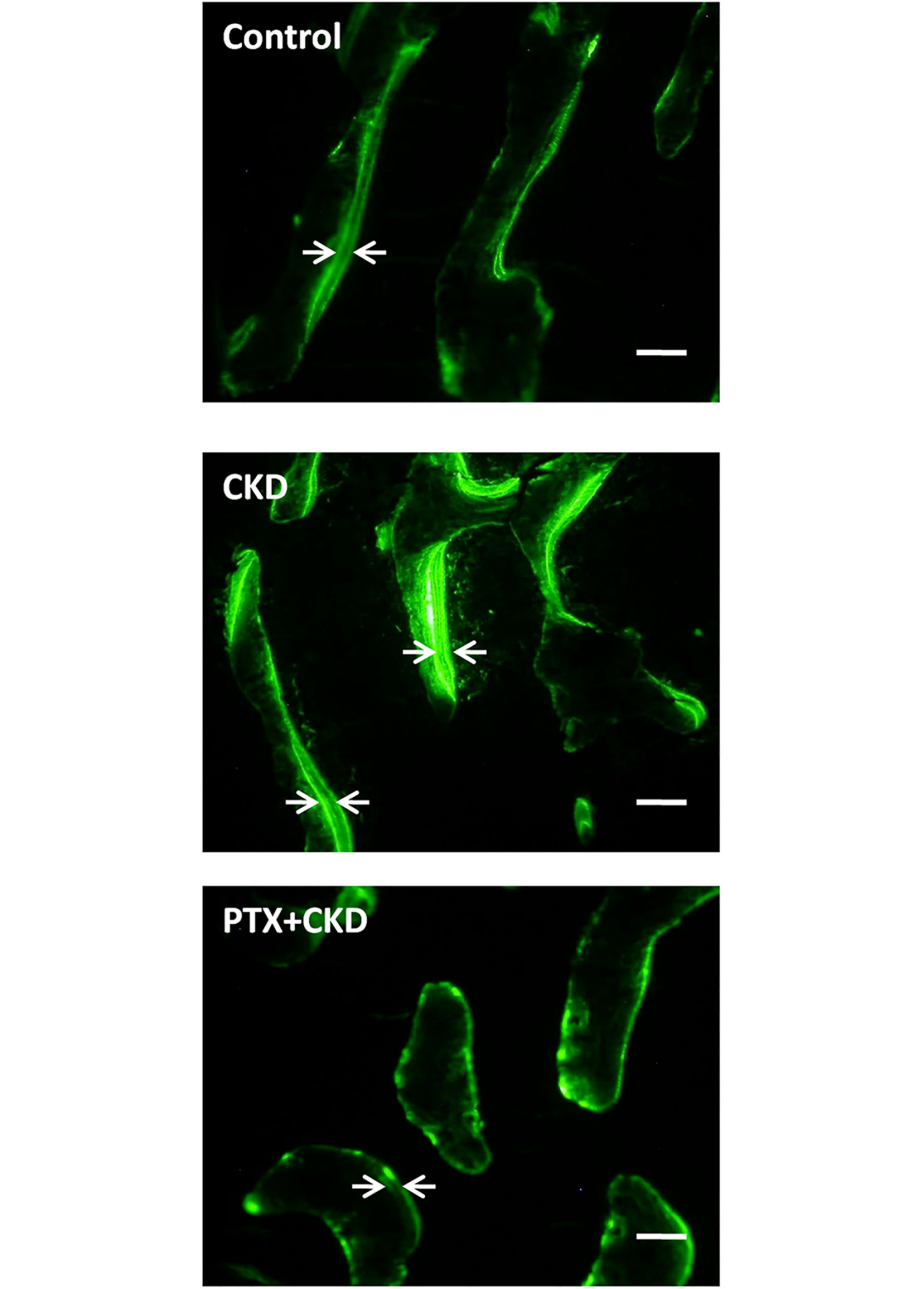
Dynamic bone histomorphometry with calcein-fluorescent labeling. The distance between the 2 lines of calcein label is greater in the chronic kidney disease (CKD) than in the control group. The PTX and chronic kidney disease (PTX+CKD) group had fewer areas with 2 labels, and the distance between the 2 lines of calcein label was smaller, indicating lower bone turnover in the PTX+CKD than in the CKD group. Scale bar = 50 μm.

Evaluation of dynamic histomorphometry (Table 1 and Fig 2) showed that the mean percent mineralized bone surface was significantly lower in the PTX+CKD than the CKD group. Mean MAR and mean BFR were significantly different among the three groups, both were highest in the CKD and lowest in the PTX+CKD groups. Following parallel evaluation of serum bone resorption marker levels and bone formation rates, the PTX+CKD group was denominated as having low bone turnover, while the CKD group had high bone turnover. OMT used as a parameter to measure mineralization rates, was not different in among the groups.

### Correlation Study and Regression Analyses

The Pearson’s correlations between various serum biomarkers were analyzed (Table 2). Serum FGF-23 levels correlated with serum intact PTH, CTX, calcium, and phosphate levels. Serum 1,25-(OH)_2_D_3_ values correlated with serum intact PTH, osteocalcin, and CTX levels. There was also an association between serum values of intact PTH and CTX. Serum calcium levels also correlated positively with serum intact PTH levels and negatively with serum phosphate levels. In addition, serum phosphate values also correlated with serum intact PTH and osteocalcin values.

**Table 2:** Pearson’scorrelation coefficients of relationships between serum biomarkers values.

		intact PTH	FGF23	ALP	Osteocalcin	1,25(OH)_2_D_3_	CTX	Calcium	Phosphate
intact PTH	*r*		.726**	-.290	-.001	-.546**	.654**	.641**	-.58**
	p		<.001	.151	.99	.004	<.001	<.001	.002
FGF-23	*r*	.726**		-.239	-.128	-.266	.647**	.718**	-.511**
	p	<.001		.240	.554	.19	<.001	<.001	.008
ALP	*r*	-.290	-.239		.091	.001	.061	-.129	-.004
	p	.15	.24		.66	.99	.77	.53	.99
Osteocalcin	*r*	-.001	-.128	.091		-.506**	.203	-.312	.464*
	p	.99	.54	.66		.008	.32	.12	.02
1,25(OH)_2_D_3_	*r*	-.546**	-.266	.001	-.506**		-.699**	-.029	.003
	p	.004	.19	.99	.008		<.001	.89	.88
CTX	*r*	.654**	.647**	.061	.203	-.699**		.340	-.316
	p	<.001	<.001	.77	.32	<.001		.09	.115
Calcium	*r*	.641**	.718**	-.129	-.312	-.029	.340		-.782**
	p	<.001	<.001	.53	.12	.89	.09		<.001
Phosphate	*r*	-.58**	-.511**	-.004	.464*	.03	-.316	-.782**	
	p	.002	.008	.99	.02	.88	.115	<.001	

Abbreviations are defined in Table 1

*r*, correlation coefficient; p, level of significance

**Correlation was significant at the 0.01 level

*Correlation was significant at the 0.05 level

Univariate linear regression was used to analyze between serum biomarkers and bone histomorphometric parameters (Table 3). Bone formation rate and MS/BS were associated with serum values of intact PTH, FGF-23, CTX, calcium, and phosphate levels. MAR was associated with circulating concentrations of intact PTH, FGF-23, 1,25-(OH)_2_D_3_, CTX, calcium and phosphate. Bone volume ratio correlated with circulating concentrations of intact PTH, FGF-23, CTX, calcium, and phosphate. Bone trabecular thickness was associated with serum FGF-23 and CTX levels. The osteoid parameters OV/BV, OS/BS and O.Th correlated with serum levels of intact PTH, FGF-23, and CTX. In addition, O.Th also correlated with serum calcium levels. The linear regression analysis in this study did not demonstrate any association between biochemical parameters and OMT, used to assess bone mineralization rates.

**Table 3:** Univariate linear regression analyses indicating relationships between serum biomarkers and bone histomorphometric parameters. Bone histomorphometric parameters values as dependent variables.

		BFR	BV/TV	thickness	O.Th	OMT
intact PTH	*β*	.714	-.657	-.360	.489	-.18
	p	<.001	<.001	NS	.011	NS
FGF-23	*β*	.707	-.737	-.526	.508	-.021
	p	<.001	<.001	.006	.008	NS
ALP	*β*	-.157	.227	.181	.023	.019
	p	NS	NS	NS	NS	NS
Osteocalcin	*β*	.094	.310	.131	-.005	-.075
	p	NS	NS	NS	NS	NS
1,25(OH)_2_D_3_	*β*	-.317	.079	.208	-.368	.128
	p	NS	NS	NS	NS	NS
CTX	*β*	.637	-.491	-.41	.555	.031
	p	<.001	.011	.037	.003	NS
Calcium	*β*	.663	-.683	-.254	.420	-.262
	p	.001	<.001	NS	.033	NS
Phosphate	*β*	-.521	.555	.058	-.307	.381
	p	.006	.003	NS	NS	NS

Abbreviations: intact PTH, intact parathyroid hormone; FGF-23, Fibroblast growth factor 23; ALP, alkaline phosphatase; CTX, collagen type I C-telopeptide; BFR, bone formation rate; MS/BS, percent mineralized bone surface; MAR, mineral apposition rate; BV/TV, bone volume ratio; O.Th, osteoid thickness; OMT, osteoid maturation time

β, regression coefficient; p, levels of significance; NS, no significant

Stepwise multiple linear regression analyses were performed to investigate any potential independent relationships between biomarkers and bone histomorphometric parameters. Serum biomarkers including intact PTH, FGF-23, ALP, osteocalcin, 1,25-(OH)_2_D_3_, CTX, calcium, and phosphate were used as independent variables. Bone histomorphometric parameters were considered dependent variables (Table 4). Serum intact PTH was an independent predictor of bone formation rate and MS/BS. Circulating calcium and 1,25-(OH)_2_D_3_ values correlated with MAR. Levels of FGF-23 were independently associated with bone volume parameters: bone volume and bone trabecular thickness. CTX was associated with osteoid parameters. No biochemical parameters could predict OMT in the present study (Table 4).

**Table 4:** Stepwise multiple linear regression analyses: bone histomorphometric parameters as individual dependent variables. The probability of entry was 0.05 and removal was 0.1 during stepwise analyses.

		BFR	BV/TV	thickness	O.Th	OMT
Adjusted *r* ^*2*^		.489	.524	.246	.279	NS
intact PTH	*β*	.714	-.257	.046	.220	-
	p	<.001	.206	.861	.338	
FGF-23	*β*	.399	-.737	-.526	.256	-
	p	.053	<.001	.006	.259	
ALP	*β*	.054	.019	.100	-.011	-
	p	.727	.899	.587	.951	
Osteocalcin	*β*	.095	.216	-.116	-.122	-
	p	.519	.122	.520	.493	
1,25(OH)_2_D_3_	*β*	.104	-.125	.073	.038	-
	p	.553	.392	.694	.877	
CTX	*β*	.297	-.025	-.120	.555	-
	p	.119	.892	.608	.003	
Ca	*β*	.349	-.317	.254	.262	-
	p	.059	.112	.319	.151	
P	*β*	-.161	.242	-.285	-.146	-
	p	.372	.134	.162	.425	

NS, not significant; other abbreviations are defined in Table 3

β, partial correlation coefficient; p, levels of significance

Stepwise multivariate regression analysis did not display any independent association between independent variables and osteoid maturation time (OMT) in the present study.

## Discussion

In the present study, the PTX+CKD and the CKD groups had similar CKD severity; the PTX+CKD group had hypoparathyroidism and the CKD group had secondary hyperparathyroidism (Fig 1). The PTX+CKD group had a low bone formation rate, low serum bone resorption marker values, and low serum FGF-23 levels; conversely, in the CKD group, there was a higher bone formation rate, higher serum bone resorption marker values, and higher serum FGF-23 levels (Table 1 and Fig 1). Stepwise multivariate regression analysis demonstrated that serum FGF-23 independently correlated with bone structure histomorphometric parameters: bone volume and bone trabecular thickness (Table 4). These data were consistent with the hypothesis that FGF-23 correlated with bone histomorphometry, more specifically with bone volume in CKD. Instead, intact PTH was independently associated with bone formation rate and CTX correlated with osteoid parameters.

The hormone PTH has been reported to stimulate FGF-23 secretion in osteocytes [29–31] and to regulate FGF-23 levels *in vivo* [32,33]. In CKD, secondary hyperparathyroidism induces high levels of serum FGF-23 [31], and PTX has shown to decrease elevated FGF-23 levels in advanced secondary hyperparathyroidism [22,34] in PTX + CKD rats (Fig 1). Accordingly, in the present study we show that serum FGF-23 levels correlated with serum PTH levels (Table 2).

Phosphate balance is regulated by both PTH and FGF-23. When the serum phosphate level is elevated, PTH and FGF-23 increase the fractional excretion of phosphate by acting on the proximal renal tubules. In CKD, the decline in renal function leads to phosphate retention. FGF-23 serum levels increase and may correlate with total phosphate load in CKD. However, in the PTX+CKD rats in our study, a low mean serum FGF-23 level was noted despite hyperphosphatemia, which was likely related to low mean circulating PTH levels (Fig 1). Thus, in this PTX+CKD group with hypoparathyroidism and low FGF-23 levels, phosphaturia had not increased [32], although the CKD group had increased fractional excretion of phosphate (as expected in CKD) [12]. Therefore, from our data it appeared that both low PTH and FGF-23 levels had limited phosphate excretion in the PTX+CKD rat model. Other animal studies with hypoparathyroidism or calcium deficiency [20,32] also demonstrated that serum phosphate values are inversely correlated with serum FGF-23 values.

FGF-23 interacts with vitamin-D and serum calcium. FGF-23 inhibits renal production of the active form of vitamin-D [35], which increases calcium absorption in the intestine. In addition to diminished renal functional mass, the decrease circulating vitamin-D concentrations in CKD patients is caused by the increase in FGF-23 levels and activity [36]. Calcium positively correlates with FGF-23 under hypocalcemia [20, 37]. In a study in which rats fed a low-calcium and low-vitamin D diet, severe hypocalcemia resulted in the decline of FGF-23 levels although serum PTH values were elevated [20]. Hypocalcemia induced by PTX might also contribute to the low circulating FGF-23 levels observed in our PTX+CKD rats. Under severe hypocalcemia, the drop in circulating FGF-23 may raise active form of vitamin D levels and rescue serum calcium levels. In our PTX + CKD rat model, serum calcium levels were all initially less than 6 mg/dL after PTX; however, at the time of euthanization, the mean serum calcium levels rose to 7 mg/dL. Accompanied by low FGF-23 levels in PTX+ CKD animals, 1,25-(OH)_2_D_3_ tended to increase exponentially compared to CKD rats. The phenomenon might be related to physiological adaption to severe hypocalcemia and prevention of further adverse effects to other tissues [37].

Osteodystrophy is a feature of CKD. In the present study, the CKD rats had a high bone turnover associated with secondary hyperparathyroidism (Table 1 and Fig 1) [38, 39]. The PTX+CKD group had a lower bone formation rate because PTX resulted in lower levels of PTH, a hormone necessary for bone cell differentiation. The PTX+CKD group had a concurrent reduction in bone resorption marker levels compared to the CKD group. With regards to the reduced bone formation rate and bone resorption rate [27], the PTX+CKD group was considered as having low bone turnover in this experimental model [22]. PTH modulates bone cells including the activity of osteocytes, osteoblasts, and osteoclasts. The net influence of PTH on bone metabolism depends on which effect predominates [40,41].

While FGF-23 is mainly produced by osteocytes, it may be associated with bone formation. In our study, FGF-23 levels showed a positive linear association with bone formation rate (Table 3), but in multiple regression analysis, FGF-23 was not an independent predictor of bone formation. Instead, the bone formation rate could be determined by PTH (Table 4). The relationship between bone formation rate and FGF-23 might be secondary to the PTH effect (Table 2). Serum levels of bone non-collagen synthetic markers, serum ALP and osteocalcin are generally used to evaluate bone turnover in CKD [42]. However, after PTX in patients with CKD, bone synthesis markers may increase for a period instead of declining [43–46] and lower bone formation rate occurs at this time [22]. The dissociated change between the bone formation rate and bone synthesis markers was also demonstrated in a study comparing bone histomorphometry before and after PTX in patients with secondary hyperparathyroidism [43]. Serum ALP values increased but the bone resorption reaction decreased after surgery. The number of osteocytes decreased and the presence of empty lacunae increased. The presence of two distinct tetracycline labeled areas could not be distinguished [43]. Instead, mineralization occurred in the osteocyte-canalicular system rather than at the mineralization front. This phenomenon may explain why bone formation rates, calculated using fluorescent labels at the mineralization front, could not positively correlate with ALP and osteocalcin after PTX in our PTX+CKD animals (Table 4).

Both intermittent and continuous treatment with PTH have been reported to increase bone formation rates in animals, while bone volume could be increased by intermittent supplement but decreased by continuous supplement [40]. Overall, bone volume is determined by the net effect of bone anabolism and catabolism [15]. Secondary hyperparathyroidism in CKD is similar to that observed with persistent exposure to PTH. High serum levels of resorption marker, CTX, were observed in our CKD rats (Table 1) and they had high catabolism rate and low bone volume parameters. Increased bone mineral density after PTX can be seen both in patients with primary hyperparathyroidism and secondary hyperparathyroidism related to CKD [47] as bone resorption decreases [43]. Our PTX+CKD rats were observed with highest bone volume parameters. Osteoid thickness decreased after PTX, while osteoid volume and osteoid surface did not differ in our rats. Osteoid parameters correlated with CTX, which presumably indicated that as more collagen cumulates, more degraded products could be generated. Parameters of mineralization assessment such as osteoid maturation time did not differ among the three groups (Table 1).

Osteocytes participate in bone remodeling by interacting with osteoblasts and osteoclasts, or by secreting systemic regulators that control mineral metabolism [7,41]. According to bone-renal axis in normal physiology, FGF-23 induces phosphaturia when the body is exposed to phosphate load. The increase in FGF-23 also inhibits renal synthesis of 1,25-(OH)_2_D by suppressing vitamin D-activating enzyme 1α-hydroxylase [36]. FGF-23 affects mineral metabolism by coordinating bone mineralization. Increased bone volume may up-regulate FGF-23 to excrete excess phosphate through the kidney and further suppress bone mineralization. Administration of bone formation promoter or anti-resorption agents affected bone metabolism and serum FGF-23 levels in one animal study [15]. The authors showed that supplementation with PTH forced an increase in the bone formation rate. The requirement for minerals increased at this phase and FGF-23 secretion was decreased in order to reduce mineral loss. When osteoprotegerin (OPG) was administered, the resorption of bone declined and bone volume increased as a primary effect. Consequently, the bone formation rate slowed down to prevent further increases in bone volume. The overall mineral requirement decreased and FGF-23 secretion was enhanced.

However, the normal physiology of the bone-renal axis is disrupted in CKD. With renal impairment, the retention of phosphate burden persistently induces PTH and FGF-23 expression. As a result, the physiological interplay regulating FGF-23, PTH, and bone metabolism in CKD may differ from those with normal renal function. In a large prospective case-cohort study, the data showed that higher levels of serum FGF-23 were associated with fracture risk in elderly men with decreased renal function but not in those without renal impairment [48]. How mineral imbalance, hormone dysregulation, or other pathophysiology in CKD affects the interplay between FGF-23 and bone metabolism requires further study. In contrast, FGF-23 was shown to inhibit bone matrix mineralization in *in vitro* studies [49,50]. A negative association between FGF-23 and bone mineral density or bone volume parameters was shown in patients with CKD [51], while in some previous studies, this relationship was not observed [52]. The authors concluded that these divergent findings might be related to different measurement sites or to the method selected to detect bone density [52]. In addition, the role played by FGF-23 in phosphate regulation and bone metabolism is likely different in diabetes mellitus patients from those without diabetes mellitus [53–55]. Therefore, the numbers of diabetes mellitus patients included in the above studies could have affected the final results. The relationship between plasma FGF-23 values and bone histomorphometric parameters in pediatric and young adult CKD patients had been previously reported in series studies from the same group [56–58]. In the children who received peritoneal dialysis supplied with oral calcitriol, serum FGF-23 independently correlated with osteoid parameters and osteoid maturation time [57]. However, in young patients with CKD stage 2–5 without dialysis, circulating FGF-23 was not consistent with the above and poorly predicted the bone formation rate. Instead, serum PTH values were independently associated with increased osteoid accumulation and circulating phosphate concentrations independently correlated with osteoid accumulation and osteoid maturation time [58]. The authors concluded that renal FGF-23 excretion might obscure the relationship between FGF-23 and bone histomorphometric variables in predialysis CKD patients. This may explain why no correlation was observed between our FGF-23 values and the osteoid parameters. In addition, the variables included in our stepwise analysis might have influenced the final results. In our study, serum CTX was analyzed and associated with the osteoid parameters after stepwise analysis. Conversely, FGF-23 negatively correlated with bone volume in the above-mentioned pediatric patients on peritoneal dialysis in bivariate analysis [57], while this relationship was not seen in young CKD patients without dialysis [58]. The bone volumes of these CKD patients were either normal or increased although most had high serum PTH and FGF-23 levels. The bone growth-inducing properties of other endogenous circulating growth hormones in these young patients and may have obscured the association between FGF-23 and bone volume parameters that was observed in our animals.

PTX resulted in an extremely high phosphate burden in the PTX+CKD group. Interestingly, FGF-23 decreased rather than increased in this experimental model. Since serum FGF-23 values were still independently correlated with bone volume, FGF-23 might exert specific actions on bone that are independent of its effects on bone mineralization. This should be confirmed with further experiments. Our study demonstrated the relationship between FGF-23, other biochemical markers, and bone histomorphometric parameters with the intention to provide a better understanding of bone metabolism in CKD. Limitations of the present study include the absence of data regarding potential of cause and effect relationships linking biomarkers and bone parameters. In the future, the experiment that treated with various concentration of FGF-23 in FGF-23 knock-out + CKD animal is a way to evaluate the sole effect of FGF-23 on bone. In addition, our study was based on animal models instead of humans. Further animal or clinical studies should take into consideration other etiologies, such as diabetes mellitus, which lead to low turnover bone in CKD.

## Conclusions

In conclusion, the rat model with CKD and secondary hyperparathyroidism (CKD group) had high serum FGF-23 levels and high bone turnover. Although in the presence of hyperphosphatemia, serum FGF-23 levels decreased in the CKD rat model with hypoparathyroidism (PTX+CKD group) and had low bone turnover. According to stepwise multivariate regression analysis, circulating FGF-23 levels independently correlated with bone volume. In addition, in the present study, serum PTH independently correlated with bone formation rate, and CTX was associated with osteoid parameters.
